# Connections Between Diabetes Mellitus, Other Metabolic and Endocrine Dysfunctions, and Cardiovascular Pathologies

**DOI:** 10.3390/biomedicines13081980

**Published:** 2025-08-15

**Authors:** Cristina Tudoran

**Affiliations:** 1 Department VII, Internal Medicine II, Discipline of Cardiology, University of Medicine and Pharmacy “Victor Babes” Timisoara, E. Murgu Square, Nr. 2, 300041 Timisoara, Romania; tudoran.cristina@umft.ro; 2 Centre of Molecular Research in Nephrology and Vascular Disease, University of Medicine and Pharmacy “Victor Babes” Timisoara, E. Murgu Square, Nr. 2, 300041 Timisoara, Romania; 3 County Emergency Hospital “Pius Brinzeu”, L. Rebreanu, Nr. 156, 300723 Timisoara, Romania

The incidence of diabetes mellitus (DM) has progressively increased over the last decades. It still maintains its ascending trend due to the modern people′s daily diet containing a large amount of glucose and lipids and the lack of physical activity, combined with the prolongation of the general population’s life expectancy. According to the World Health Organization, it is estimated that around 830 million people are currently suffering from DM. MS is characterized by the coexistence of metabolic and cardiovascular (CV) risk factors in the same patient, such as dyslipidemia, elevated fasting glucose level and/or insulin resistance (IR), and increased blood pressure values or even systemic hypertension (SH). DM, metabolic syndrome (MS), and obesity are leading causes of morbidity and mortality worldwide. On the other hand, the incidence of CV disease (CVD) is also growing, especially in developing countries. This trend could at least partially explain why physicians encounter more often patients with several endocrine pathologies and/or metabolic disorders and CVD.

Physicians and researchers should not underestimate the enormous impact of DM, MS, obesity, hypothyroidism, primary aldosteronism (PA), and iron deficiency on the evolution of CVD, especially if the patient also develops heart failure (HF). Special attention should be given to pregnant women, as the onset of each of these endocrine disorders during this physiologic condition could worsen the patient′s prognosis. Advances in gene regulation technology, clarification of pathophysiologic pathways in the glucose metabolism, development of new therapeutic approaches, and the use of artificial intelligence (AI) to improve diagnostic protocols open new horizons for the management of patients with various metabolic and endocrine dysfunctions, also allowing a precocious detection, even in a subclinical status, of CV alterations, thus preventing their evolution to a symptomatic disease with multiple consequences for the patient.

This Special Issue aims to highlight new insights into the significance of several risk factors (gene expression, race, ethnicity, and socioeconomic status) for the diagnosis and treatment of patients with DM and CVD and on the outcome of these individuals. The articles published in “Connections Between Diabetes Mellitus, Other Metabolic and Endocrine Dysfunctions, and Cardiovascular Pathologies” focus on three main topics: diagnostic challenges, treatment options, and uncommon associations between metabolic disorders and CVD in specific populations. The prevention and treatment of CVD during pregnancy have always raised challenges for physicians, and this Special Issue provides valuable insights for the better management of these women.

Regarding innovative diagnostic possibilities, special attention has been granted to the options offered by genetic analysis. In this regard, Stanciu et al. discuss the impact of the mammalian target of rapamycin (mTOR) dysregulation on hyperglycemia and IR. MTOR is a serine/threonine protein kinase that belongs to the phosphoinositide 3-kinases (PI3K)-related protein kinase family, exerting a key role in glucose and lipid metabolism, cell growth, survival, and proliferation. mTOR hyperactivation may cause hyperglycaemia and IR, with a higher incidence in the Western population. The inhibition of mTOR, by various drugs, determines anti-inflammatory, antioxidant, and antitumoral effects, leading to decreased levels of glucose and triglycerides, and reduction of body weight. Dietary habits, proinflammatory cytokines, increased salt intake and renin–angiotensin–aldosterone system (RAAS) dysregulation induce mTOR overactivation, favoring the development of IR and AH.

Another point of interest was the early detection and effective risk assessment of DM and CVD, considering the increasing incidence of their association with impact on patients’ outcomes. Advances in oculomics, exploring the relationship between retinal microvascular changes and systemic vascular health, offer a promising non-invasive approach for assessing CV risk. Retinal fundus imaging and optical coherence tomography/angiography provide critical information for precocious diagnosis of retinal vascular changes (vessel caliber, tortuosity, and branching patterns). Given the large volume of data generated during routine eye exams, there is a growing need for automated tools to facilitate diagnosis and risk prediction. Artificial intelligence (AI)-driven analysis of retinal images can accurately predict CV risk factors and events, and metabolic diseases, surpassing traditional diagnostic methods in some cases. AI-generated models achieve high sensitivity and specificity, surpassing other classical diagnostic methods. This approach highlights the potential of retinal imaging as a key component in personalized medicine, enabling more precise risk assessment and earlier intervention. It not only aids in detecting vascular abnormalities that may precede CV events, but also offers a scalable, non-invasive, and cost-effective solution for widespread screening. By integrating oculomics into routine clinical practice, healthcare providers could significantly enhance early detection and management of systemic diseases, ultimately improving patient outcomes. Fundus image analysis thus represents a valuable tool in the future of precision medicine and CV health management.

Other laboratory analyses, as the triglyceride-to-high-density lipoprotein (TG/HDL) ratio, stand out as a viable option to determine early changes in lipid metabolism associated with IR, offering a cost-effective and straightforward alternative to traditional, more complex biomarkers. This research reveals the TG/HDL ratio’s predictive power across ethnicities and sexes, with specific thresholds providing greater accuracy for Caucasians, Asians, and Hispanics compared to African Americans, and for men versus women. The analysis of the TG/HDL ratio is a simple, accessible marker for IR.

The pathogenesis of diabetic kidney disease (DKD) is complex and multifactorial. It is frequently associated with different degrees of albuminuria, ranging from normoalbuminuria, microalbuminuria, to macroalbuminuria. Considering its complications and reduced number of diagnostic biomarkers, it is important to develop new biomarkers with potential roles in the early diagnosis of DKD. Glycine, tiglylglycine, kynurenic acid, and tryptophan may be considered promising biomarkers for early diagnosis of DKD and DM type 2 progression follow-up.

DM type II, along with atherosclerotic vascular disease, results in several feared complications, one of them being acute peripheral ischemia. Actual research focuses on identifying new inflammatory markers for the diagnosis, treatment of critical ischemia, and prognosis assessment. Further studies are required to define risk classes and to estimate the prognosis of critical ischemia. We also need to identify specific markers for the clinical staging of this disease in the post-revascularization phase, both in terms of surgical complications related to revascularization and of major CV events.

Another DM type 2 complication is diabetic retinopathy. The most important risk factors for the occurrence, evolution, and management of diabetic retinopathy are poor glycaemic control and longer DM duration, while the most impacting comorbidities are AH, CVD, chronic kidney disease (CKD), and dyslipidaemia. Early detection and management of systemic risk factors may avert this DM complication. However, the primary and fundamental strategy for preventing ocular complications in diabetic patients requires tight glycaemic control.

Recent data suggest that patients with DM are predisposed to develop myocardial fibrosis sooner, a contributor to adverse cardiac remodelling following acute myocardial infarction, leading to the occurrence of HF, a complex clinical syndrome caused by structural or functional dysfunction of the ventricular filling. In this regard, in the last years, new drugs have been developed, some of which are not only effective in reducing basal blood glucose levels, but they also improve the outcome of patients with HF. Other studies draw attention to the fact that most antidiabetic drugs have controversial effects on the risk of HF (thiazolidinediones, saxagliptin in the DPP-4i, and even insulin). Researchers still debate the safety of metformin in patients with HF. Emerging evidence suggests that anti-diabetic medications, such as sodium-glucose transporter 2 (SGLT2i), glucagon-like peptide-1 receptor agonists (GLP-1Ras), and dipeptidyl peptidase-4 (DPP4i), may have an important role in reducing fibrosis, improving CV outcomes, and reducing the burden of HF. SGLT2i and even GLP-1 RAs achieved positive results for HF endpoints, with SGLT2i in particular significantly reducing the composite endpoint of CV mortality and hospitalization for HF. Further understanding of the mutual pathophysiological mechanisms between HF and DM may facilitate the detection of novel therapeutic targets to improve the clinical outcome.

Another topic of this Special Issue was the diagnosis and treatment of patients with primary aldosteronism (PA) and associated comorbidities treated by general practitioners. We determined a significant association between PA and various chronic diseases, including AH, hypokalemia, hepatic steatosis, gout, CKD, obesity, and depression. Notably, PA patients exhibited a markedly higher likelihood of developing gout compared to those without PA. The findings underline the need for early detection and individualized management strategies for PA to mitigate the associated risks and improve patient outcomes.

Pregnancy is a physiologic condition that can exacerbate underlying pathologies or even cause complications endangering both mother and child. Some of these comorbidities raise diagnostic and therapeutic challenges. The pathologies debated in this Special Issue are endothelial dysfunction, SH, and hypothyroidism in pregnant women and their impact on the fetus. We also focused on methods to prevent these co-morbidities in healthy high-risk pregnant women. We demonstrated that in pregnant women with preeclampsia and pregnancy-induced hypertension, increased arterial stiffness may be involved in the pathogenesis of preterm birth, suggesting an association between fetal development and maternal endothelial dysfunction. Pulse wave analysis may be a non-invasive, reliable, clinically applicable method for assessing maternal arterial stiffness (AS). It may be more relevant to intrauterine fetal growth when associated with lower serum 25(OH)D levels. Subclinical hypothyroidism treated with levothyroxine in women with gestational DM does not increase the risk of gestational hypertensive disorders but is associated with an increased risk of prematurity. Petre et al. state that physical training, coordinated by a medical team consisting of an obstetrician, a physiotherapist, and a cardiologist, decreases systolic and diastolic blood pressure values and heart rate in healthy pregnant women. Strictly supervised physical training may reduce pregnancy-induced SH. Pulse wave velocity is also recommended for screening of elevated AS during pregnancy. Additionally, regular exercise in healthy pregnant women decreases AS, suggesting that it may help to prevent endothelial dysfunction, AS, and SH during pregnancy.

Iron deficiency (ID) is a common condition in patients with acute coronary syndrome. Although its influence on short-term follow-up appears to be uncertain, ID seems to be independently associated with a worse prognosis during the mid- to long-term follow-up of the patient. Transferrin saturation, contrary to ferritin, is not upregulated during acute conditions, which may be crucial for the non-invasive diagnosis of ID. Transferrin saturation appears to have a greater predictive value on death and other adverse events compared to ferritin or serum iron concentration, but there are new, promising, precise markers employed for non-invasive ID diagnosis in clinical practice. Iron supplementation in patients with acute coronary syndrome may have a beneficial effect on myocardial healing and left ventricular remodeling after myocardial infarction, but further research, especially on human models, is needed to support this theory.

Dumitrescu et al. point out that disadvantaged populations exhibit a significantly higher prevalence of DM, impaired glucose regulation, SH, and dyslipidemia. Another risk factor for CVD is the type of obesity, especially important in patients with DM type 1. In men, central adiposity, assessed by total, trunk, and android fat mass, is associated with higher glucose and lower glucose variability. In women, gynoid fat mass, but not other body composition parameters, is related to hyperglycemia. The observed associations of body weight and body composition with the features of daily glucose dynamics may be mediated by insulin sensitivity.

Cosoreanu et al. studied the prevalence of CV risk factors, CVD, and microvascular complications among the Roma population, underscoring the importance of considering ethnic disparities in approaching healthcare management strategies. Regarding the anthropometric and paraclinical assessments, her data evidenced different characteristics in the Roma compared to a corresponding group of non-Roma, highlighting the importance of tailoring healthcare interventions to the specific needs and cultural context of this ethnic minority.

Fukuda et al. highlight the particularities of IR in the Japanese population. In these individuals, the age-related high prevalence of DM and impaired insulin tolerance is associated with impaired insulin secretion rather than IR.

A specific situation arose at the end of 2019, when the coronavirus disease (COVID-19) resulted in a worldwide pandemic. New data regarding the multisystemic effects of the severe acute respiratory syndrome coronavirus 2 (SARS-CoV-2) emerged. Several studies determined that COVID-19 has negative effects on the glycemic control in DM and non-DM patients. Predicting post-COVID-19 diabetes is crucial for enhancing patient care and public health. Our research investigates the role of metabolic factors in predicting glycemic outcomes in patients recovering from moderate to severe COVID-19. Conventional diabetes risk factors, including body mass index and lipid profiles, showed low predictive power for post-COVID-19 glycemia. This Special Issue highlights the critical role of metabolic and inflammatory pathways in managing glycemic control in COVID-19 patients. Markers such as blood glucose, Homeostasis Model Assessment of Insulin Resistance, and high-sensitivity C-reactive protein are significant predictors of blood glucose levels, while the triglyceride/glucose index appears less helpful in this context. Early, targeted interventions based on these markers can improve patient outcomes and reduce the risk of post-COVID-19 complications, such as DM.

This compilation of articles highlights the importance of a precocious diagnosis and assessment of the risk profile in patients with metabolic diseases and CVD, especially HF, as well as in specific physiological conditions (pregnant women) and special populations (the Roma population, native Japanese, and people living in developing countries). Special interest should be given to pregnant women with subclinical hypothyroidism or with vitamin D deficiency and pre-eclampsia. Further studies are required to determine the importance of specific genetic and environmental factors and lifestyle changes on CV risk profiles in subjects with metabolic disorders for targeted interventions addressed to these high-risk populations. Continued research efforts in this area are essential for improving our understanding and refining treatment strategies to optimize patients’ outcomes.

